# Research on Online Education Resources Recommendation Based on Deep Learning

**DOI:** 10.1155/2022/3674271

**Published:** 2022-09-09

**Authors:** Xu Wang

**Affiliations:** School of Government, Sun Yat-sen University, Guangzhou 510275, Guangdong, China

## Abstract

For the problem of knowledge overload in the process of online learning and the traditional algorithm's poor recommendation accuracy and real-time performance in the massive educational resources, a deep learning-based recommendation model for online educational resources is proposed. First, attribute features of learners and learning resources are extracted, and then text features of learning resources are extracted, and attention fusion of features at multiple different scales is performed using a multiscale fusion strategy. Finally, the fused features are used as input to the multilayer perceptron to train the classification model. Through testing a variety of educational resources, it is verified that the model in this paper has better real-time performance while maintaining high detection accuracy and outperforms the mainstream comparison model in several indexes, which have a certain application value. It provides a new way of thinking for educational platforms to build real-time educational resource recommendations.

## 1. Introduction

The rapid development of science and technology has greatly facilitated people's lives. As the Internet becomes more and more widely used in various fields, it has led to a geometric increase in the amount of information available in all sectors of society [1]. Although people have different areas of concern and are confronted with more or less information, in general, these large amounts of information make people be in a sea of information, for example, film and television resource sites such as Aiki and Youku store large amounts of videos, movies, and other resources. Taobao, Jingdong, and other business sites also store tens of thousands of products, stores, and other resources. Sites such as Amazon have many kinds of book resources; sites such as MOOC also have a variety of online educational information as well as learning resources [2]. Faced with such a huge web resource, it is difficult to discover and find the information you care about. Moreover, the same information affects the value of different people in different ways. Positive information enables people to learn news and information about the world and the city faster and also enables people to quickly grasp information that is beneficial to their development, speeding up the pace of development, and promoting social development and progress [3]. Existing methods to help people find positive information quickly and accurately mainly use search algorithms, which present people with only the sorted results of the search, leading to the “information overload problem” [4, 5]. To help people accurately get valuable information of interest, resource recommendation systems based on recommendation algorithms have emerged. Traditional recommendation algorithms include collaborative filtering recommendation algorithms, content-based recommendation algorithms, etc. Among them, the collaborative filtering recommendation algorithm tends to ignore some more detailed issues, each individual is different, and it is difficult for the collaborative filtering algorithm to comprehensively calculate the personalized features generated by these differences, which is very likely to cause poor user experience.

Video as a major information carrier has greatly enriched the information content. In recent years, online learning has gradually become one of the options for global learning education, and a large number of massive online open courses have gradually entered people's vision as a new form of education, among which Coursera, edX, and Udacity are the mainstream online learning platforms [6]. MOOC provides learners with ways and channels to acquire knowledge flexibly beyond the conditions of time, space, and location, enabling the development of mobile Internet technology while effectively promoting the development of online education. As a result, online users need to face new challenges such as resource “overload” and “disorientation” due to the huge amount of learning content and the rapid iteration of resources [7]. Due to the rapid growth of teaching video data, how to improve the efficiency of users to find videos has become one of the main problems that online learning platforms need to solve now. In order to improve the efficiency of finding instructional videos, existing methods mainly use tag indexing, search engines, and other methods. The widespread use of methods such as tag indexing and search engines has improved the efficiency of finding videos to a certain extent. However, this method is single and static feedback to the user, cannot accurately grasp the user's needs and interests, and the mode is fixed and inefficient.

The rapid development of the Internet has led to the development of education. The online platform, as a shared, inclusive, and open information-gathering center, stores a large number of educational resources. With the advancement of the domestic network environment, network bandwidth, and mobile communication technology, students have not only been satisfied with classroom and book knowledge but also have gradually moved toward the Internet and are on a massive growth trend. Students can browse and study the resources they need at will with the help of the online platform, and the cost and threshold of acquiring knowledge are greatly reduced. In recent years, the Ministry of Education also vigorously develops and promotes the development of online education on the Internet, which has a positive role in facing the current problem of uneven distribution of educational learning resources, vigorously promoting the development of education, promoting the sharing of educational resources, establishing a network of high-quality curriculum resources, unifying the knowledge of educational resources, and promoting the sustainable development of education and resources [8]. It also emphasizes accelerating the popular application of online learning spaces, improving the cloud service system for educational resources, and further strengthening the construction of online open courses.

Nowadays, each education platform vigorously develops online education on the Internet, but most education platforms only provide learning resource retrieval functions, making it difficult to find matching, appropriate documents, and resources, which require students to spend a certain amount of time to select quality and appropriate learning resources for themselves, which is a huge waste of time. Thus, how to build an educational resource recommendation system that can take into account learners' differences and provide both retrieval functions and accurate recommendations of learning resources to students according to their learning information and personality characteristics, so that they can acquire knowledge more effectively and improve their learning efficiency is an urgent need to be addressed in the current education system.

## 2. Related Works

User behavior is one of the most important ways to understand user intent, so it is often used to describe user needs by analyzing user behavior. In the era of Big Data, data analysis methods are widely used in Internet data analysis tasks. In the educational video, resource recommendation algorithm based on user behavior, user attitude, viewing intention, and user interests and needs are visualized by analyzing user behavior in the process of watching videos to facilitate better services for users [9].

### 2.1. Status of Research on Personalized Models

Modeling the learner means describing the personalized characteristics of the learner formally so that the system can understand the learner timely and accurately, and thus provide personalized services to the learner. The features selected for the learner personalization model cover learners' natural attributes, cognitive abilities, knowledge levels, learning styles, learning preferences, learning abilities, learning intentions, learning interests, and learning habits.

In recent years, studies related to learner models have mainly focused on theoretical research. The factors included in learner models are comprehensive and involve various aspects, but they are difficult to apply in practice development and are not very operable. While recent models have focused on a particular characteristic of the learner and conducted practice development and applied research. In terms of knowledge models, the literature [10] proposes a learner knowledge model based on the connections between domain concepts. The literature [11] proposes a learner model that automatically models the relationships between different domain concepts. The literature [12] uses conceptual networks to model learners' knowledge [6]. The literature [13] models learners' knowledge based on representations of integration skills, skill-to-skill relationships, and skill-to-item relationships. In terms of cognitive ability, the literature [14] proposes a learner model for estimating cognitive deficits in learners with dyslexia or dyscalculia. In terms of learning styles, the current mainstream learning style theories include VARK and Felder–Silverman learning style theories, and the literature [15] also explores the application of Felder–Silverman learning styles in the design of online courses, and a linear function system model has been used to develop a course analysis tool based on course feedback results. The tool allows instructors to determine how well a course supports a particular learning style based on the Felder–Silverman model.

Generally speaking, there has been a lot of research work on learner models, with many scholars contributing from all aspects, from the field of education to the field of computing. However, most of the current learner models suffer from the unsystematic, incomplete, and subjective acquisition of feature values, poor computability, and research that is mostly at the theoretical level. So based on learners' behavioral data on online education platforms, this topic proposes a learner personalization model with multisource information fusion and it also focuses on the interest model and knowledge model of learners and gives the formal representation and calculation methods of the interest model of learners and the knowledge model of learners.

### 2.2. Status of Research on Recommendation Algorithms

Personalized recommendation algorithm has become hot research in educational resource management platform, and the existing recommendation algorithm mainly includes collaborative filtering algorithm based, collaborative filtering recommendation algorithm based, etc. For example, in [16], user preferences are modeled by analyzing user ratings and annotations to help users filter out useless e-mails, which marks the emergence of “collaborative filtering” of recommendation systems. In [17], the concept of a “recommender system” is proposed based on “collaborative filtering” to recommend feature items that may be of interest to users. The literature [18] constructs item similarity networks based on users' browsing records to predict users' interest preferences and find items that users may be interested in for recommendation. In the literature [19], a clustered collaborative filtering recommendation method based on a trust relationship is proposed to address the problem of inaccuracy and small coverage of recommendation algorithms under clustering after data dimensionality reduction. First, based on the clustering algorithm of singular value decomposition, the degree of trust between targets is analyzed by the relationship matrix to find the circle of trust. Second, a parse rating supplement algorithm is proposed to generate dense user rating profiles, which alleviates the sparseness and cold start problems to some extent. Finally, it combines real-time updated user ratings and collaborative filtering algorithms to find recommendation items. On top of that, the recommendation algorithms of many social circle network applications are also based on collaborative filtering recommendation algorithms, which analyze users' preferences and find similar user recommendations. The literature [20] proposes a video recommendation algorithm based on complex relational graphs. The algorithm calculates the similarity between two users, based on the relationship graph between the user and the project node, and it generates predictions based on the calculated similarity values, evaluates the similarity of the recommended items to the content of the user's history, and recommends them to the user. Based on a collaborative filtering algorithm with the addition of covariance, regularization, and matrix decomposition, literature [21] mines and analyzes the user history from the perspective of users and items, improves the recommendation constraints, and selects the decision formula flexibly. Meanwhile, the computational complexity and spatial complexity of the algorithm are optimized to improve the recommendation accuracy. The literature [22] proposes a video recommendation method based on content similarity and social networks. The method first understands the video content, second analyzes the set of users from which the video originates to determine the social relevance of the video, and finally recommends based on user circle similarity and content similarity. The literature [23] proposes a collaborative filtering recommendation algorithm based on Slope One for user features, which first, analyzes user features to cluster users, second, predicts the ratings of users within the same group based on similarity, and finally finds recommendable items by collaborative filtering. The literature [24] improved the similarity calculation method based on the collaborative item filtering recommendation algorithm to improve the accuracy of prediction and classification, starting from the scoring of items and item structure. The literature [25] proposes a dynamic user interest recommendation algorithm that combines a semantic understanding of evaluation content. The algorithm first understands and models the semantics of the evaluated content. Second, it statistically analyzes users' preferences for different videos to identify their interests. By adding a time window mechanism to dynamically analyze changes in user interests, recommendations are made based on user interests.

In summary, recommendation algorithms have achieved good results in theoretical research, while personalized recommendation systems have been well developed in practical applications. However, most recommendation methods use learner preference information obtained by weighted averaging of course embedding vectors and do not consider the effect of different courses on learner preference effects. So this paper proposes a deep learning-based course recommendation method, which builds deep recommendation models based on online education resource data and further improves personalized recommendations by capturing features between learners and courses at a deep level.

## 3. Methodology

**Figure 1:**
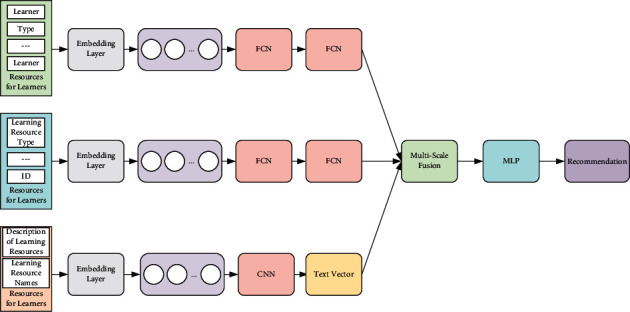
The framework of the recommendation model with the deep learning.

Figure 1 gives the framework of the online education resource recommendation model based on deep learning in this paper, which includes four parts: attribute feature extraction of learners and learning resources; text feature extraction of learning resources; multiscale feature fusion; and recommendation generation.

### 3.1. Attribute Feature Extraction of Learners and Learning Resources

First, preprocessing the course description text content can better capture the semantic information between sentences, that is, the course description text information is represented in the form of an index vector, and then the index vector is converted into a word distribution representation. And it is worth noting that these word index sequences are padded with “0,” and since the input to the network is a fixed-length word vector. Therefore, the first “0” in the word representation vector can be ignored, and the “1” is the index that uniquely identifies the word, and then, the word “index” vector is input to the embedding layer of the neural network, and the embedding layer is used to find the embedding representation corresponding to each word, and the specific embedding is based on the word2vec method of the word vector representation. By encapsulating these word vectors into a matrix via word2vec, the resulting matrix is an embedding representation of the text content of a particular course description, and further correlations between courses are captured based on this embedding matrix.

The learner information and learning resource attribute information are input into the model of this paper to obtain the attribute characteristics of learners and learning resources. Assume that the learner's attribute is *x*={*x*_1_, *x*_2_, ⋯, *x*_*n*_}, where *x*_*i*_ denotes one of the learner attributes, such as learner ID. The attributes of a learning resource can be denoted as *y*={*y*_1_, *y*_2_, ⋯, *y*_*n*_} and *y*_*i*_ denote one of the learning resource attributes, such as the learning resource ID. Then the attributes of learners and learning resources are input to the embedding layer to obtain the learner and learning resource attribute feature vectors ${\bar{x}}_{i}$${\bar{y}}_{i}$, which are calculated as shown in the following equations:

(1) $$\begin{array}{c} \bar{x}=f\left(w_{1}x+b_{1}\right)\text{,} \end{array}$$

(2) $$\begin{array}{c} \bar{y}=f\left(w_{2}y+b_{2}\right)\text{,} \end{array}$$

where *w*_1_ and *w*_2_ denote the weights, *b*_1_ and *b*_2_ denote the offset. *f*(·) denotes the activation function. The learner characteristics *u*_*i*_ are then obtained by fusing the individual attribute characteristics of the learner using the following equation:

(3) $$\begin{array}{c} u_{i}=Conc\left(\bar{x}\right)\text{,} \end{array}$$

where Conc(·) represents the concatenate operation.

Similarly, the attribute features *s*_*i*_ of the learning resources can be obtained and calculated as shown in the following equation

(4) $$\begin{array}{c} s_{i}=Conc\left(\bar{y}\right)\text{.} \end{array}$$

### 3.2. Text Feature Extraction of Learning Resources

**Figure 2:**
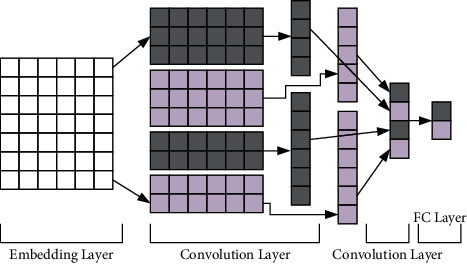
Convolution neural network model.

The convolutional neural network [26] in the model is mainly used to obtain the textual features of the learning resources from the textual information of the learning resources. The learning resource text information is first represented as a learning resource text vector by word2vec and then input to the convolutional neural network to extract features. The structure of the convolutional neural network model contains four layers: the embedding layer, the convolutional layer, the pooling layer, and the fully connected layer, as shown in Figure 2.

#### 3.2.1. Embedding Layer

In the neural network, the embedded data have low dimensionality and can map discrete sequences into continuous vectors. Here, the embedding layer is used to convert the textual information of the learning resource into an embedding matrix, where each row in the matrix is a participle element. As shown in the embedding layer in Figure 2, assuming that there are a total of 7 words, each represented by a 5-dimensional vector, a 7 × 5 dimensional matrix can be obtained, and this matrix is the equivalent of an “image” for the convolution layer to convolve. The text matrix *D* of the learning resources can be expressed as

(5) $$\begin{array}{c} D=\left[\begin{array}{c} w_{11}\cdot \cdot \cdot w_{1i}\cdot \cdot \cdot w_{1m}\\ w_{21}\cdot \cdot \cdot w_{2i}\cdot \cdot \cdot w_{2m}\\ \cdot \cdot \cdot \cdot \cdot \cdot \cdot \cdot \cdot \\ w_{n1}\cdot \cdot \cdot w_{ni}\cdot \cdot \cdot w_{nm} \end{array}\right]\text{,} \end{array}$$

where *m* represents the dimensionality of the embedding, *n* represents the number of words, and [*w*_*i*,1:*m*_] represents the vector form of the *i*-th word.

#### 3.2.2. Convolution Layer

**Figure 3:**
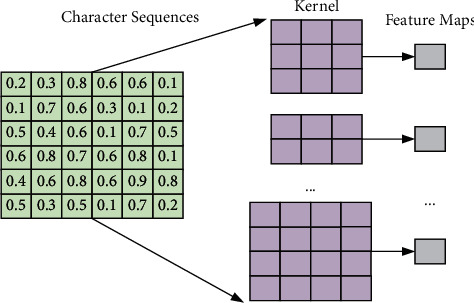
The process of the convolution operation.

Multiple convolution kernels of different sizes are used to make convolution operations on the embedding matrix, and the window size refers to how many words are covered by each convolution. To cover the whole word-embedding vector, this paper sets the convolution size in the format of the number of words × vector dimension. Here, two 3 × 5, and two 2 × 5 convolution kernels perform convolution operations on the 7 × 5 embedding matrix, respectively, and the convolution kernel of size 3 × 5 slides three words at a time, and the convolution kernel of size 2 × 5 slides two words at a time, resulting in four different feature maps. The feature map is calculated as shown in equation (6). The operation flow of the convolution kernel is shown in Figure 3.

(6) $$\begin{array}{c} F_{i}^{\prime }=f\left(D⊗F_{i}+b_{i}\right)\text{,} \end{array}$$

where ⊗ denotes the convolution calculation, *b*_*i*_ denotes the bias term, and *f*(·) is a nonlinear activation function. Nonlinear factors can be introduced into the model to solve the feature vectors that are difficult to represent in a linear model, and the Relu activation function is used in this model.

#### 3.2.3. Pooling Layer

The pooling layer is mainly used after the convolution layer to reduce the feature map dimension and the number of network parameters by a down-sampling operation. Common pooling operations include average pooling and maximum pooling. The pooling operation can ignore small variations in the feature maps and improve accuracy, while effectively avoiding the overfitting phenomenon. Suppose the feature map obtained in the *t*-th convolutional layer is *M*_*t*_={*m*_1_, *m*_2_,…, *m*_*s*_}. The maximum pooling strategy is used to extract the maximum value for *M*_*t*_, *p*_*i*_ denotes the pooling result of the *t*_*i*_ convolutional layer, and the pooling operation can be expressed as

(7) $$\begin{array}{c} p_{i}=\max\left(M_{t}\right)=\max\;\left\{m_{1},m_{2},\ldots ,m_{s}\right\}\text{.} \end{array}$$

#### 3.2.4. Fully Connected Layer

The main role of the fully connected layer is to synthesize the previously extracted feature values and output a fixed-size feature vector. Suppose there are *m* neurons in the fully connected layer, and after the Relu activation function, a fixed-size vector *s* is obtained, which is the text feature vector of the learning resource. The calculation is shown in the following equation:

(8) $$\begin{array}{c} s=\sigma \left(w_{i}p_{i}+b_{i}\right)\text{,} \end{array}$$

where *p*_*i*_ denotes the output of the learning resource text information on the pooling layer, *σ* is the Relu activation function, *w*_*i*_ denotes the weight, and *b*_*i*_ is the bias. With the above description, the CNN model constitutes a function where the input data are the textual information of the learning resource and the output result is a feature vector for each textual information. That is, the textual characteristics of the learning resources can be expressed as

(9) $$\begin{array}{c} t_{j}=CNN\left(W,Y_{j}\right)\text{,} \end{array}$$

where *W* denotes all the weights and bias variables, *Y*_*j*_ denotes the original text information of learning resource *j*, and *t*_*j*_ denotes the text feature vector of learning resource *j*.

### 3.3. Multiscale Feature Fusion

**Figure 4:**
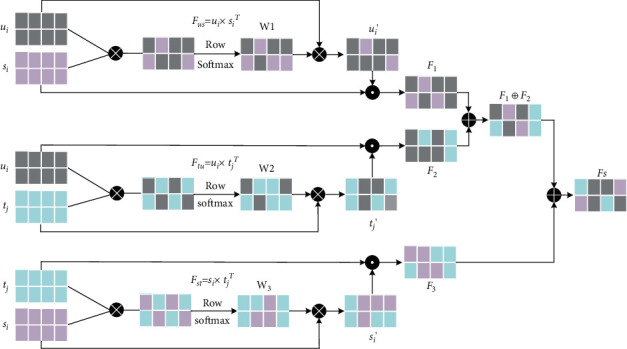
Multiscale feature fusion attention mechanism.

The proposed attention mechanism is inspired by the human visual mechanism, and the basic idea is to weaken irrelevant information and increase the attention of focused information during the operation [27]. In this paper, learner features *u*_*i*_, attribute features *s*_*j*_of learning resources, and text features *t*_*j*_ of learning resources are fused using the multiscale feature fusion attention mechanism shown in Figure 4.

First, the matching matrices *F*_*us*_, *F*_*tu*_, and *F*_*st*_ are calculated between the learner features *u*_*i*_, the attribute features *s*_*j*_ of the learning resource, and the text features *t*_*j*_ of the learning resource using the following equation:

(10) $$\begin{array}{c} \left\{\begin{array}{c} F_{us}=u_{i}\times s_{i}^{T},\\ F_{tu}=u_{i}\times t_{j}^{T},\\ F_{st}=s_{i}\times t_{j}^{T}\text{.} \end{array}\right. \end{array}$$

The attention distribution weights *w*_1_, *w*_2_, and *w*_3_, respectively, of the matching matrix are calculated by the softmax function, and multiply the weights *w* with the individual scale feature matrices to obtain the attention representation matrices *u*_*i*_′, *s*_*i*_′, *t*_*j*_′, expressed as shown in the following equation:

(11) $$\begin{array}{c} \left\{\begin{array}{c} u_{i}^{\prime }=u_{i}\times w_{1},\\ s_{i}^{\prime }=s_{i}\times w_{2},\\ t_{j}^{\prime }=t_{j}\times w_{3}\text{.} \end{array}\right. \end{array}$$

Finally, the multiplicative gating mechanism is used to multiply the attentional representation with another single-scale feature for the corresponding elements to obtain the interscale mutual attention matrices *F*_1,_*F*_2_, and *F*_3_, represented as shown in the following equation:

(12) $$\begin{array}{c} \left\{\begin{array}{c} F_{1}=u_{i}^{\prime }\cdot s_{i}\text{,}\\ F_{2}=t_{j}\cdot u_{i}\text{,}\\ F_{3}=s_{i}^{\prime }\cdot t_{j}\text{.} \end{array}\right. \end{array}$$

The joint features of *F*_1_ and *F*_2_ and *F*_3_ are operated jointly to obtain the final multiscale static fusion features, represented as shown in the following equation:

(13) $$\begin{array}{c} Fs=\left(F_{1}⊕F_{2}\right)+F_{3}\text{,} \end{array}$$

where (×) denotes matrix fork multiplication, (·) denotes matrix dot product, and ⊕ denotes Cat operation.

### 3.4. MLP Predicts Scores and Generates Recommendations

The above multiscale fused features Fs are used as the input of the multilayer perceptron to predict the scores. Here, to achieve end-to-end optimization of the model in this paper, a cross-entropy loss function is used and the weights of each layer are adjusted and determined by backpropagation. Finally, the prediction results are given quickly by mapping the activation function to the range of [0, 1]. The calculations are shown in the following equations:

(14) $$\begin{array}{c} X_{t}=wh_{t}+b\text{,} \end{array}$$

where *h*_*t*_ is the decoder output hidden vector. *X*_*t*_ is the fully connected result:

(15) $$\begin{array}{c} P\left(\left.y\right|x\right)=\frac{e^{h\left(x,y_{i}\right)}}{\overset{n}{\underset{j=1}{\sum }}e^{h\left(x,y_{i}\right)}}\text{,} \end{array}$$

where *x* is the fully connected result, *y* is the true description, and *P* is the softmax function:

(16) $$\begin{array}{c} L\left(\theta \right)=-\overset{T}{\underset{t=1}{\sum }}\log\;p\left(\left.y^{t}\right|y_{1:t-1}^{*}\right)\text{,} \end{array}$$

where *θ* denotes the model cross-entropy loss balance parameter.

## 4. Experiments

### 4.1. Experimental Environment

The experimental running environment is Ubuntu 18.04 with 128G RAM and NVIDIA Tesla T4 GPU with 32G graphics memory; PyTorch deep learning framework with GPU acceleration support is used, and the Cuda environment is NVIDIA CUDA 11.3 and cuDNN V8.2.1 deep learning acceleration library.

**Figure 5:**
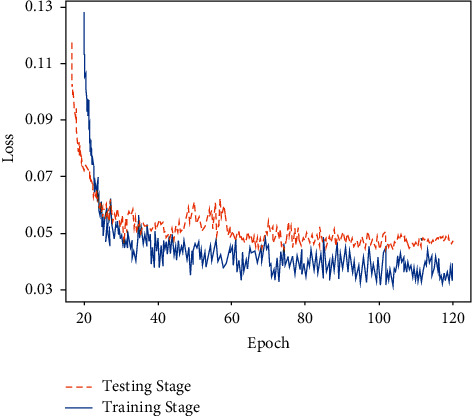
Loss curves.

The network was trained with the stochastic gradient descent algorithm SGD in the experiments, with an initialized learning rate of 0.001, a learning decay rate of 0.005, and a maximum iteration period of 3 × 10^4^, and the model training loss curve is shown in Figure 5. The momentum factor is set to 0.9. In addition, to solve the model overfitting problem, dropout is introduced to remove some neurons randomly, and dropout takes the value of 0.5 in this paper. As can be seen from Figure 5, when the number of model iterations Epoch is 60, the loss curves of both the training and test sets smooth out, and the loss values are below 0.04, indicating that the model has converged.

### 4.2. Experimental Results and Analysis

#### 4.2.1. Comparison of the Recommended Effect

In this paper, the traditional collaborative filtering recommendation algorithm (User-CF) [28] based on user play records, the collaborative filtering recommendation algorithm (FCNN-CF, Fully Connected Neural Network) [29] for determining user preferences, and the recommendation model in this paper is compared on the same data set. And accuracy rate AR (Accuracy Rate), precision rate PR (Precision Rate, PR), recall rate RR (Recall Rate, RR), and *F*1 value was used as evaluation metrics.

**Figure 6:**
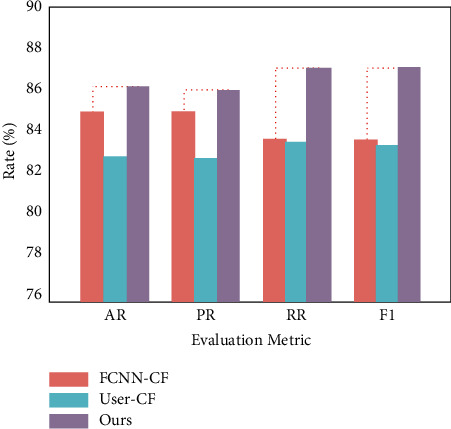
Comparison of the recommended performance of different models.

From Figure 6, it can be seen that the model in this paper has improved by 2.01% (84.6% vs. 86.3%) and 4.48% (82.6% vs. 86.3%) in terms of accuracy compared to the comparison models FCNN-CF and User-CF recommendation models, respectively. In terms of precision, the models in this paper improved by 1.30% (84.9% vs. 86.1%) and 4.74% (82.2% vs. 86.1%), respectively; in terms of recall, the models in this paper improved by 4.31% (83.5% vs. 87.1%) and 4.69% (83.2% vs. 87.1%), respectively. In terms of *F*1, the models in this paper improved by in terms of *F*1, the model improved by 4.32% (83.4% vs. 87.0%) and 4.95% (82.9% vs. 87.0%), respectively. The above experimental results verify that the model in this paper has better recommendation performance, mainly because this paper uses multiscale features for attention fusion of features from different sources, which effectively suppresses edge information and focuses on strong features that are better for model classification performance.

#### 4.2.2. Robustness Testing

**Figure 7:**
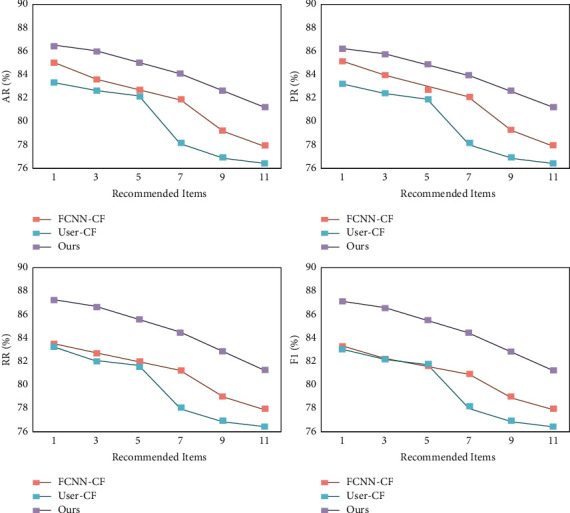
Different number of recommended items.

In addition, to verify the stability of the model in this paper, the recommended performance on several different types of items was verified separately, and the experimental results are shown in Figure 7. It can be seen that with the increase in the number of recommended projects, the advantage of this paper is obvious. Particularly, when the number of recommendations reaches 11, the model in this paper is higher than 80% for both AR, PR, RR, and *F*1 metrics. The above data further verify the robustness of the model in this paper.

#### 4.2.3. Recommended Real-Time Testing

**Figure 8:**
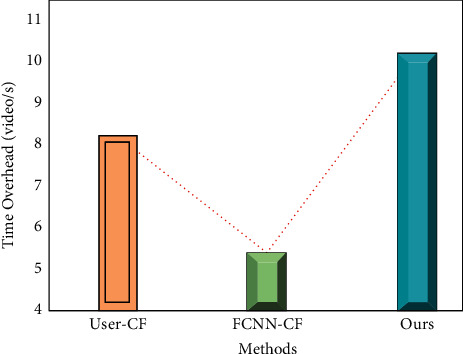
Comparison of the recommendation success rate of different models.

To verify the real-time performance of the recommended model in this paper, tests are conducted on the same data and environment. The results are shown in Figure 8. It can be seen that the model in this paper can achieve the recommended rate of 10 video/s, the FCNN-CF model can achieve the recommended rate of 5 video/s, and the User-CF model can achieve the recommended rate of 8 video/s. The above data show that the model in this paper can achieve a better recommendation success rate, mainly because this paper takes into account the personalized service of users and combines the attribute characteristics of learners and learning resources for a personalized recommendation.

## 5. Conclusion

Although recommendation algorithms are widely used, there are still various shortcomings in the educational system that need to be continued to be improved. In this paper, we address the problem of low precision and low real-time accuracy of users' access to valuable information from massive educational resources and combine the multiscale fusion technology in deep learning to make recommendations according to users' personalized requirements and present various educational resources required by users in real time. The effectiveness of personalized recommendations in this paper is verified by conducting various experimental tests on the self-built dataset.

## Data Availability

The data used to support the findings of this study are available from the corresponding author upon request.
